# Psoriatic disease and body composition: A systematic review and narrative synthesis

**DOI:** 10.1371/journal.pone.0237598

**Published:** 2020-08-13

**Authors:** Tim Blake, Nicola J. Gullick, Charles E. Hutchinson, Thomas M. Barber

**Affiliations:** 1 Rheumatology Department, University Hospitals Coventry and Warwickshire NHS Trust, Coventry, United Kingdom; 2 Warwick Medical School, University of Warwick, Coventry, United Kingdom; 3 Department of Imaging, University Hospitals Coventry and Warwickshire NHS Trust, Coventry, United Kingdom; 4 Warwickshire Institute for the Study of Diabetes, Endocrinology and Metabolism, University Hospitals Coventry and Warwickshire NHS Trust, Coventry, United Kingdom; VU University Medical Center, NETHERLANDS

## Abstract

**Background:**

Obesity is a leading comorbidity in psoriatic disease, including both psoriasis (PsO) and psoriatic arthritis (PsA), and is associated with adverse metabolic and cardiovascular (CV) outcomes. Anthropometric parameters, such as weight, body mass index (BMI) and waist-to-hip ratio, have been extensively reported in psoriatic disease. However, the associations of body composition and fat distribution with psoriasis have not yet been fully defined.

**Objectives:**

To identify whether patients with psoriatic disease, including psoriatic arthritis, have altered body composition compared with the general population, and to review existing modalities for the assessment of body composition.

**Methods:**

Electronic searches of the literature were conducted in PubMed, Medline (Ovid^®^), Embase (Ovid^®^), Cochrane Central Register of Controlled Trials (CENTRAL) and Google Scholar. Titles and abstracts were reviewed by two authors independently against a set of prespecified inclusion/exclusion criteria. The research question was answered with a systematic literature review and results were summarized narratively.

**Results:**

Twenty-five full text articles met the inclusion criteria and were included in the final narrative analysis. The studies were of heterogeneous design and used a range of objective measures to assess body composition, including simple anthropometric measures, bioimpedance analysis (BIA), dual energy X-ray absorptiometry (DXA) and computed tomography (CT). Few studies met all the quality assessment criteria. Clinical heterogeneity prevented meta-analysis.

**Conclusions:**

Patients with psoriatic disease reveal defined body composition changes that are independent of obesity and the customary metabolic syndrome, including higher overall body fat, visceral fat and sarcopenia. These findings emphasize that patients with psoriatic disease should be screened for abnormal adipose effects beyond their weight and body mass index (BMI). Our findings show that the last decade has seen an exciting expansion of research interest in the development and validation of new modalities for the assessment of body composition. There is no consensus on the optimal assessment method of body composition for this diverse group; hence there is a need for validation of existing modalities and standardization of assessment tools.

## Introduction

**Fig 1:**
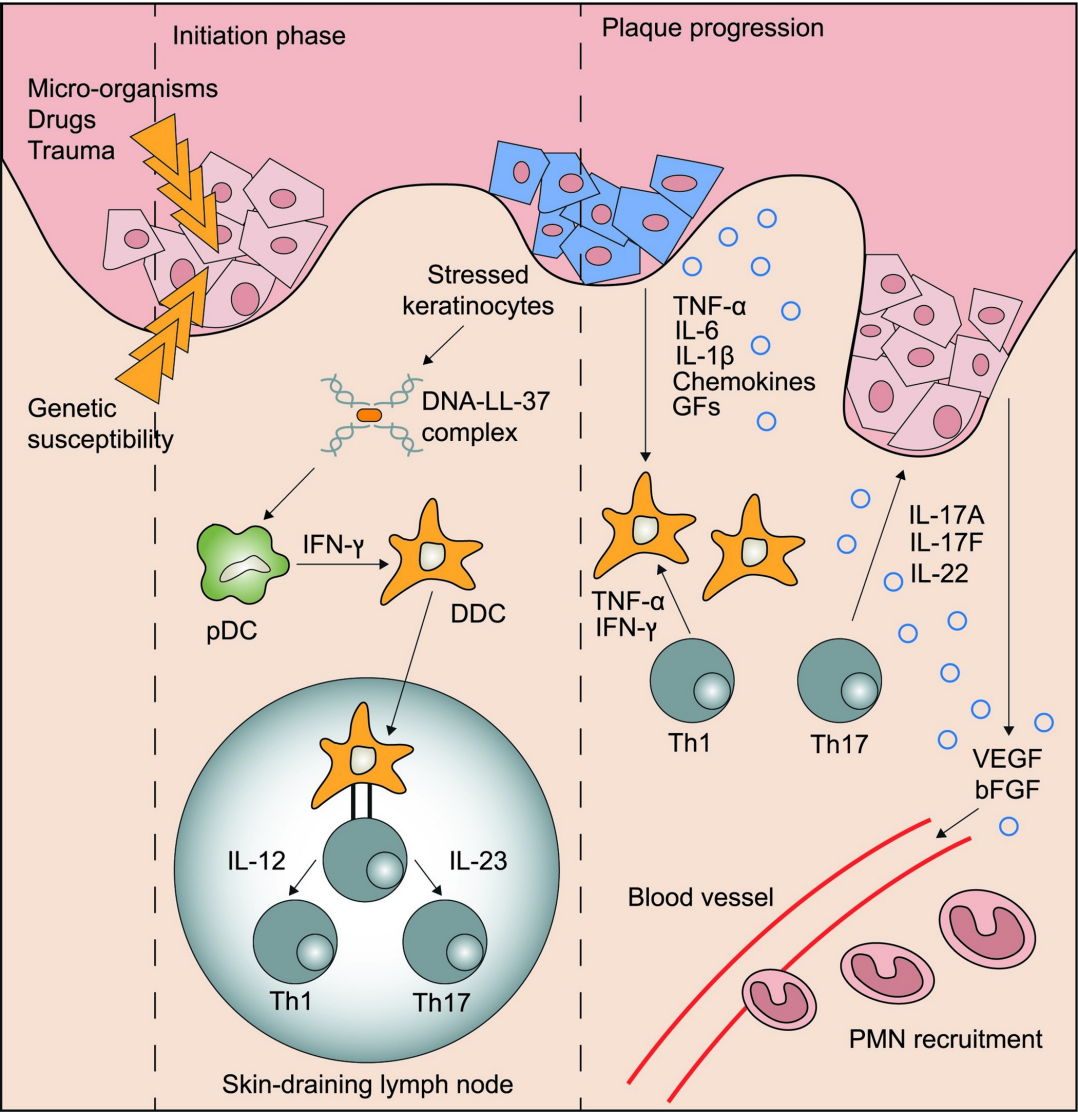
Immunopathogenesis of psoriasis. Cell subsets and cytokine signaling pathways implicated in the pathogenesis of psoriasis. Damage to the epidermis triggers release of antimicrobial peptides including LL-37, which complexes with self-DNA released from cellular membrane rupture. DNA-LL-37 complexes are autoantigens of psoriasis, which are taken up by dendritic cells, resulting in IL-12 and IL-23 production. The IL-23/Th17 axis actuates a feedforward loop that favors keratinocyte proliferation, ultimately forming a psoriatic plaque. bFGF: basic fibroblast growth factor; DDC: dermal dendritic cell; GFs: growth factors; pDC: plasmacytoid dendritic cell; VEGF: vascular endothelial growth factor.

Psoriasis (PsO) is an immune-mediated chronic inflammatory disease affecting the skin, entheses and joints, with an estimated prevalence in adults ranging from 0.5 to 11.4% and in children from 0 to 1.4% [1]. It is characterized at the skin level by infiltration of immune cells in the dermis and epidermis, vascular proliferation and atypical keratinocyte differentiation. Pathogenesis is complex and thought to result from the interaction between genetic, environmental and immunologic factors; key players in this process are T cells, antigen presenting cells, keratinocytes, Langerhans' cells, macrophages, natural killer cells, as well as multiple cytokines and growth factors including vascular endothelial growth factor and keratinocyte growth factor [2]. In recent years, the mindset has shifted from one of a Th1-driven immune response with IFN-γ and IL-12 as the signature cytokines to one in which the IL-23/Th17 axis with IL-17, IL-21 and IL-22 plays a more central role [3]. Fig 1 illustrates the immunopathogenesis of psoriasis.

**Fig 2:**
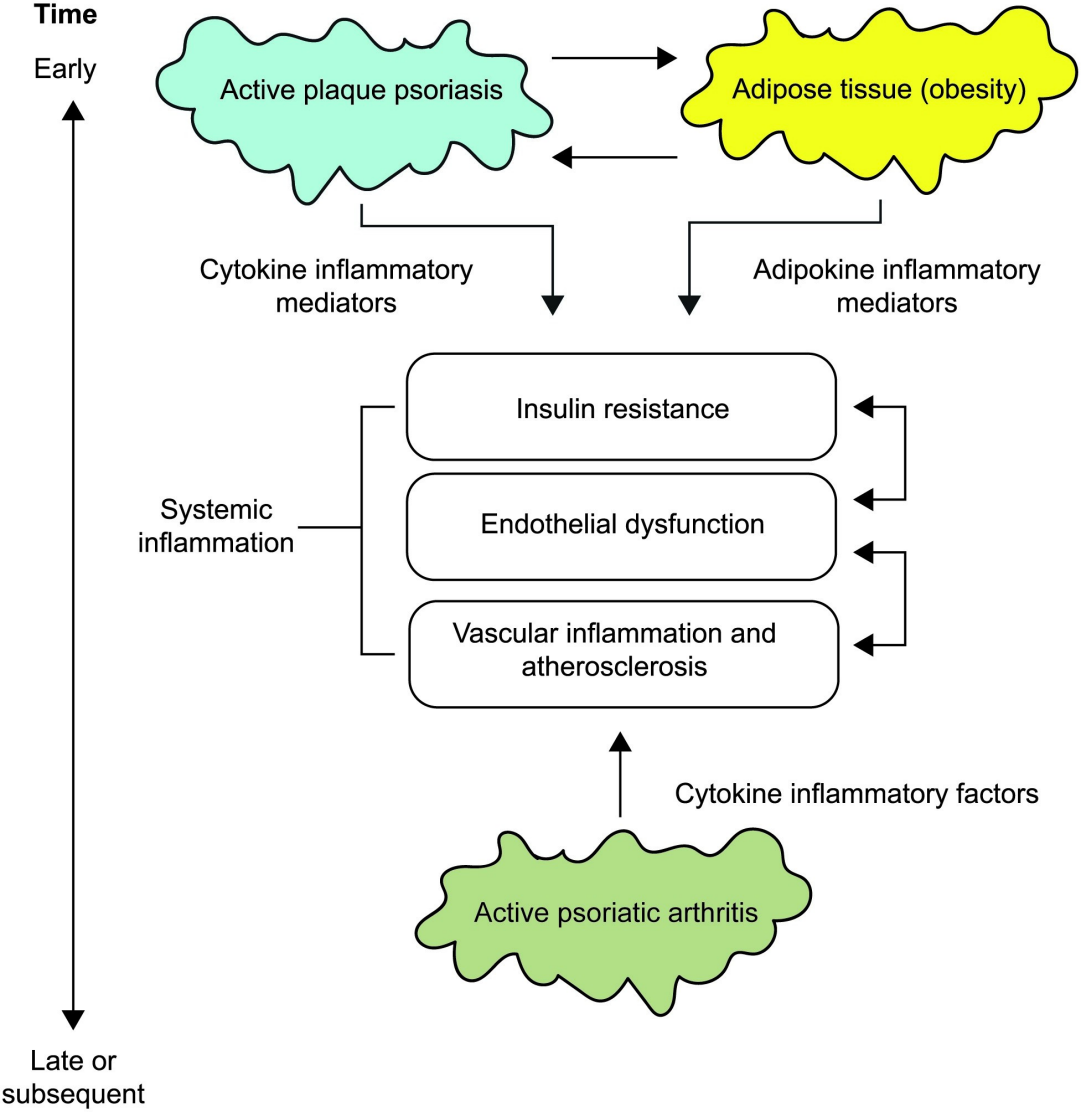
Psoriatic disease and systemic inflammation. The ‘psoriatic march’: it portrays the causal link between psoriasis as a systemic inflammatory phenomenon and cardiovascular disease. Obesity, a known risk factor for psoriasis, is capable of inducing a low-grade systemic inflammatory state. Continuous effective systemic therapy may halt the ‘psoriatic march’ through interference with insulin resistance and endothelial dysfunction.

There is increasing recognition that psoriasis is more than skin deep and has important consequences beyond the skin. Proinflammatory molecules released during chronic inflammation are implicated in certain co-morbidities, such as obesity, hypertension, diabetes mellitus, cardiovascular disease and depression. Paradoxically, epidemiologic evidence infers that obesity, via pro-inflammatory pathways, predisposes to both development and progression of psoriasis [4]. This association is shared with metabolic syndrome (MetS), not least the increased prevalence of cardiovascular risk factors and the ensuing cardiovascular morbidity [5–9]. Recent studies have suggested that adipokines, such as leptin, adiponectin and resistin, produced by adipocytes and dysregulated in obesity and MetS, are the linchpins of this metabolic association and the so called ‘psoriatic march’: a concept of how severe psoriasis can drive cardiovascular comorbidity [10]. They have been shown to contribute independently to the adverse cardiovascular outcomes in patients with PsO and can be viewed as biomarkers of obesity-related inflammation and cardiovascular risk [4]. With this is mind, one should consider adipose tissue as an endocrine organ that has the capabilities, through local and systemic factors, to induce a low-level inflammatory state. Moreover, specific IL-17-secreting Th17 cells and IL-22-secreting Th22 cells have been seen to infiltrate the adipose tissue and represent local mediators of inflammation and insulin resistance, something that is being studied in more detail. Fig 2 represents this relationship between psoriasis, obesity and metabolic dysfunction.

Sustained inflammation due to psoriatic disease also leads to loss of muscle mass and muscle weakness termed sarcopenia; however, the reasons for these muscle changes in the context of inflammation are multifactorial and difficult to define. External factors, such as aging, decreased physical activity secondary to stiffness and pain, hormonal changes and disturbances in protein metabolism are likely to exert considerable influence on this process [11].

Despite this knowledge about the associations between psoriatic disease, adipogenesis and metabolic dysfunction, there is less emphasis on body mass alterations and distribution between separate internal compartments: fat-free tissue (lean body mass), extracellular water and adipose tissue. Body composition, a measure of lean and fat mass proportions, provides a useful indicator of metabolic health [12], and its measurement in psoriatic disease could provide a useful insight into cardiometabolic risk, something that could well influence patient management.

Our principal aim was to determine the evidence for the association between psoriasis and abnormal patterns of fat (including preponderance of visceral fat content) and muscle distribution. We also explored and compared different modalities used for assessment of fat distribution. We hope that this analysis may guide future clinical decision making with respect to risk assessment, screening and management of psoriatic patients in day-to-day practice, encouraging more individualized care and leading to better patient outcomes.

## Methods

This systematic review was performed following methodology recommended by the Cochrane Collaboration and is reported according to the Preferred Reporting Items for Systematic Reviews and Meta-analyses (PRISMA) guidelines [13, 14].

### Eligibility criteria

The following inclusion and exclusion criteria were applied.

Inclusion criteria:

Types of studies

- Publication date 2004 –present (inclusive)
- Studies from any geographical location
- English language
- Published studies (including conference papers)
- Grey literature (not published in a peer-reviewed journal) including dissertations/theses
- Any quantitative study (RCT, non-RCT, observational, cohort, case control)
- Studies using qualitative methods of analysis (to describe patterns or themes raised by studies) seeking to understand body composition phenotypes of psoriatic disease. This includes original qualitative studies, studies involving secondary analysis of data, and a qualitative study as part of a mixed methods study e.g. the study also has a quantitative component
- The reference lists of the final articles included for full data extraction were hand searched for further relevant articles

Types of participants

- Adults (>18 years)
- Diagnosed with psoriasis, psoriatic disease or psoriatic arthropathy/arthritis
- Being treated in any ‘usual care’ setting: primary; secondary; tertiary care, e.g. in the hospital, hospice, community, home or rehabilitation
- Receiving care typical for that geographical location

Types of outcome measures

- Understanding and learning about body composition and metabolic phenotypes including measures of body fat and lean mass in men and women with psoriatic disease
- To explore the metabolic, anthropometric and biochemical indices of psoriatic disease

Exclusion criteria:

- Non-English language
- Published pre-2004
- Any study where quantitative or qualitative data are not analyzed i.e. uninterpreted data; case reports; any editorial, meta-analysis or review (systematic, narrative, qualitative)
- Treatment guidelines and pathways
- Commentary articles, written to convey opinion or stimulate research/discussion, with no research component

Types of participants

- Children or Young adults (<18 years)
- Diagnosis of Spondyloarthritis or Spondyloarthropathy, Ankylosing Spondylitis, other immune-mediated inflammatory diseases; studies that do not focus on psoriatic disease per se

Types of outcome measures

- Anything that is not concerned with demonstrating either a metabolic, structural or functional relationship to psoriatic disease; studies focusing on individual components of metabolic syndrome (diabetes mellitus, hypertension, hyperlipidemia or fatty liver disease) and any association with psoriatic disease

### Databases and searches

The search strategy was developed by two of the authors (TB and NG) and a librarian. Database searches were performed in PubMed, Medline (Ovid^®^), Embase (Ovid^®^), Cochrane Central Register of Controlled Trials (CENTRAL) and Google Scholar for reports published between 2004 to November 2019 using a sensitive methodologic filter for studies. A fifteen-year time frame was chosen to capture the most contemporary and innovative studies in this area. Search terms and criteria are shown in Table 1. Search results were combined into a single Endnote file and duplicates removed.

**Table 1:** Search terms.

**Psoriatic disease**
psoriatic arthritis.mp. or exp Arthritis, Psoriatic/
psoriatic arthropathy.mp. or exp Arthritis, Psoriatic/
exp Psoriasis/ or psoriatic disease.mp.
exp Spondylarthritis/ or Spondyloarthropathies/ or spondyloarthritis.mp.
**Metabolic composition**
metabolic syndrome.mp. or exp Metabolic Syndrome/
exp Body Weight/ or metabolic syndrome.mp. or exp Blood Glucose/ or exp Obesity/ or exp Lipids/ or exp Insulin/ or exp Metabolic Syndrome/ or exp Glucose Tolerance Test/
body composition.mp. or exp Body Composition/
exp Exercise/ or sarcopenia.mp. or exp Muscle, Skeletal/ or exp SARCOPENIA/ or exp Muscular Atrophy/ or exp Muscle Proteins/
sarcopaenia.mp.
**Limits**
English language
Humans
Last 15 years of publication

### Study selection

Two authors (TB, NG) independently reviewed all titles/abstracts in the web-based software platform Covidence [15] and selected articles for full-text review. Discrepancies were resolved by consensus.

### Data extraction and quality assessment

The data extraction tool was designed by TB and included a Quality Assessment incorporating a Critical Appraisal Skills Programme (CASP) checklist [16] specific for the study design. The checklists included criteria for information and selection bias addressing the following domains: participants, controls, measurement of variables, statistical power, confounding factors and applicability of results. All included studies were appraised with the Risk Of Bias In Non-randomized Studies of Interventions assessment tool (ROBINS-I) [17].

## Results

### Search results

**Fig 3:**
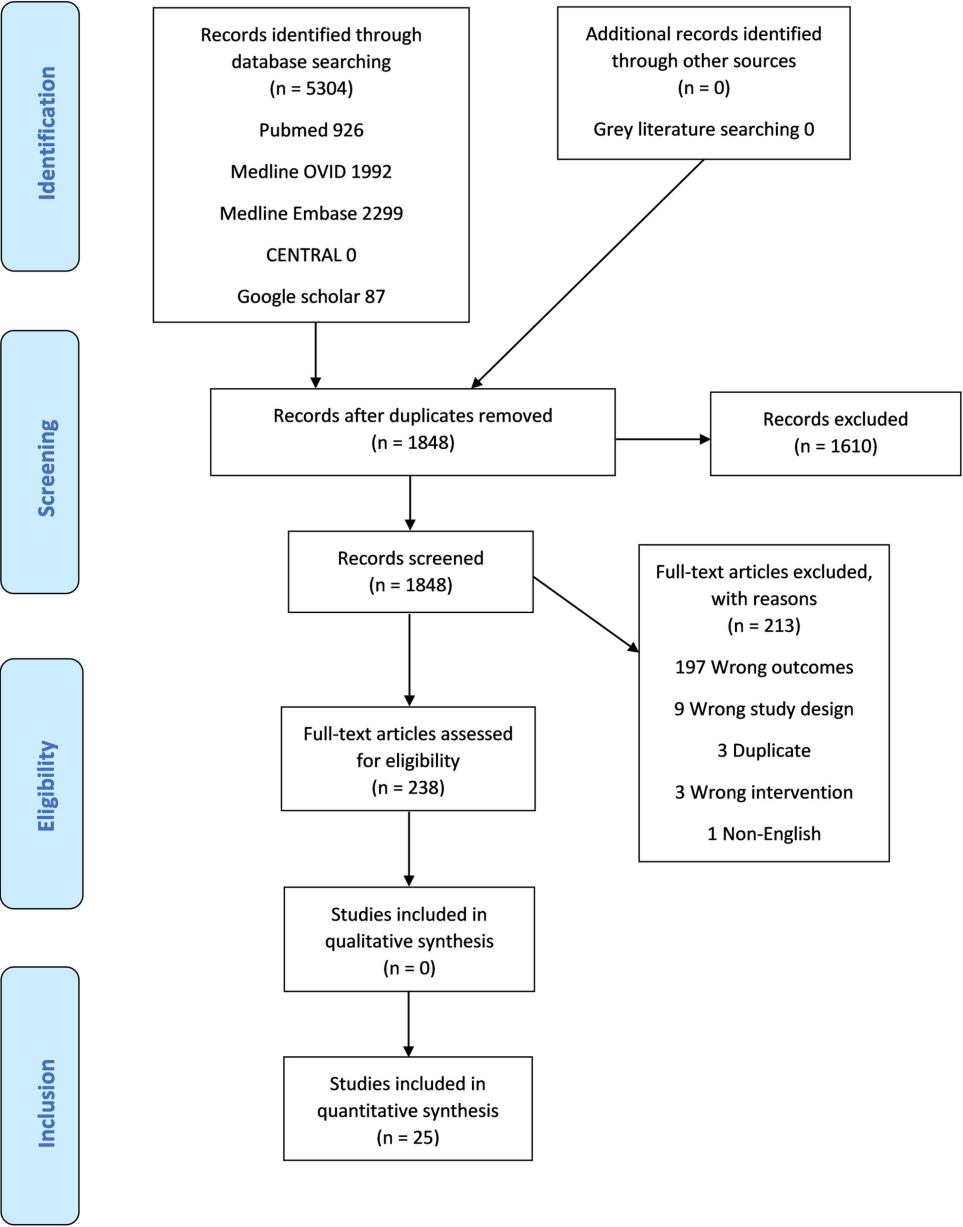
PRISMA flowchart.

The initial search yielded 5304 titles. After removing duplicates, 1848 titles and abstracts were screened, then 238 retained for full text review. A further 213 studies were excluded due to: incorrect outcomes (197), incorrect study design (9), duplicate material (3), incorrect intervention (3) and non-English language (1). A total of 25 studies from 25 publications met the inclusion criteria (Fig 3) with differing methodology: 1 randomized control trial, 1 case-control study, 19 cross-sectional and 4 prospective cohorts. A search of the grey literature (the first 100 articles sorted by relevance on Google Scholar) retrieved no additional studies. A summary of the included studies is shown in Table 2.

**Table 2:** Meta-summary of included studies.

Author, year and country	Study design	No. participants Psoriasis Controls		Outcomes
Aguiar et al., 2014, Portugal	Cross-sectional	60	60	MMI: muscle mass/height^2^ using Lee’s equation
Akyildiz et al., 2014, Turkey	Cross-sectional	31	32	EFT: β = Standardized regression coefficient
Andreevskaia et al., 2013, Belgium	Cross-sectional	117	0	Body fat (%) using a new computerized conversational system
Balci et al., 2010, Turkey	Cross-sectional	46	46	VFA: cm^2^, VFA/SFA ratio
Balci et al., 2014, Turkey	Cross-sectional	38	38	EFA: cm^2^
Barone et al., 2018, Italy	Cross-sectional	168	0	MMI by BIA
Barrea et al., 2015, Italy	Cross-sectional	62	62	FM from BIA: %
Barrea et al., 2016, Italy	Cross-sectional	180	180	Data on phase angle (PhA), a direct measure by BIA: PhA (°)
De La Brassinne et al., 2016, Belgium	Cross-sectional	552	0	Mean body fat percentage (%) using an automatic conversational system
Demirel et al., 2013, Turkey	Cross-sectional	30	30	Body composition (%) established with BIA
Diniz Mdos et al., 2016, Brazil	Cross-sectional	42	41	Percentage of total fat by DXA: (%)
Engin et al., 2014, Turkey	Case-control	242	110	Body composition values (%) measured using the Tanita SC-330 Body Composition Analyzer^®^
Ferguson et al., 2019, UK	Prospective cohort	60	0	Visceral, subcutaneous, and liver fat percentage on MRI
Galluzzo et al., 2017, Italy	Cross-sectional	164	0	Body composition (single-frequency 50 kHz) by BIA
Galluzzo et al., 2018, Italy	Prospective cohort	53	0	Body composition (%) by BIA
Ganguly et al., 2016, India	Cross-sectional	40	42	LAP index, a measure of visceral fat
Gonul et al., 2017, Turkey	Cross-sectional	41	41	Body fat distribution, particularly visceral adipose tissue by ultrasonography: abdominal fat index (AFI = Pmax/Smin ratio)
Kofoed et al., 2012, Denmark	Cross-sectional	9	0	Lean body mass and fat mass (kg) by DXA
Krajewska-Wlodarczyk et al., 2017, Poland	Cross-sectional	51	44	Total fat mass (kg) Appendicular Lean Mass index (kg/m^2^) using BIA Skeletal Muscle Index (%) using BIA
Leite et al., 2018, Brazil	Randomised control trial	97	NR	Body fat mass (%) by DXA
Renzo et al., 2011, Italy	Prospective cohort	40	0	Fat mass (kg and %) by DXA
Romani, Caixas et al., 2012, Spain	Cross-sectional	50	50	Body fat (%) using BIA
Romani, Caixas, Ceperuelo et al., 2012, Spain	Cross-sectional	50	50	Body fat content as calculated by BIA
Tournadre et al., 2017, France	Cross-sectional	148	0	Total lean mass (kg), SMI, (kg/m^2^), Total fat mass (kg), FMI, Overfat (Body fat percentage >27% for men and 38% for women) by DXA
Toussirot et al., 2019, France	Prospective cohort	52 PsO 52 PsA	52	Lean mass, fat mass and fat distribution (android/gynoid regions and visceral fat) by DXA

AFI: abdominal fat index; ALM: appendicular lean mass; BIA: bioelectric impedance analysis; CT: computed tomography; DXA: dual energy X-ray absorptiometry; EFA: epicardial fat area; EFT: epicardial fat thickness; FM: fat mass; FMI: fat muscle index; LAP: lipid accumulation product; MMI: mean muscle index; MRI: magnetic resonance imaging; NR = not recorded; SMI: skeletal muscle index; SFA: subcutaneous fat area; VFA: visceral fat area.

### Population sampled

Twenty-five studies represented a population of 2468 psoriatic patients from 10 countries: Belgium, Brazil, Denmark, France, Italy, Poland, Portugal, Spain, Turkey and UK. Demographics are shown in Table 3. Studies did not differentiate outcomes according to subtypes of psoriatic disease; however, five of the studies focused purely on psoriatic arthritis [18–22]. Only four studies [21–24] reported on the racial and ethnic groups of participants. A control group was assessed in 10 studies [18, 20, 21, 23, 25–30], and this information was missing in another study [19]. None of the studies reported on socio-economic status, including employment or educational attainment of subjects.

**Table 3:** Demographic data of populations.

Author, year and country	Age of Psoriasis group, years (mean ± SD unless stated)	Sex, female %	Ethnicity	Duration, years (mean ± SD unless stated)	Disease type	Participant selection
Aguiar et al., 2014, Portugal	45.5 ± 13.4	51.7	Caucasian	10.9 ± 11.6	PsA	PsA according to CASPAR criteria. Controls recruited from primary healthcare.
Akyildiz et al., 2014, Turkey	42.0 ± 11.1	54.8	ND	17.1 ± 10.5	PsO	Subjects attending the outpatient dermatology clinic. Clinical examination in keeping with psoriasis.
Andreevskaia et al., 2013, Belgium	48.8 ± 14.9	49.5	ND	ND	PsO	Subjects attending a new outpatient psoriasis center recruited at random of their consultation.
Balci et al., 2010, Turkey	39.5 ± 14.2	37.0	ND	11.0 ± 8.3	PsO	Patients attending outpatient dermatology clinic. Controls age and sex matched.
Balci et al., 2014, Turkey	42.2 ± 15.0	31.6	ND	14.9 ± 9.5	PsO	Patients attending outpatient dermatology clinic. Controls age and sex matched.
Barone et al., 2018, Italy	55.3 ± 9.1	84.2	Caucasian	11.1 ± 8.1	PsA	Patients with a clinical diagnosis of RA, SA, and PsA enrolled
Barrea et al., 2015, Italy	50.2 ± 10.5	30.0	ND	ND	PsO	Patients with a diagnosis of mild-to-severe psoriasis lasting for at least 6 months were enrolled, while patients with pustular, erythrodermic or arthropathic psoriasis or receiving any systemic treatment for psoriasis including acitretin, ciclosporin, methotrexate, phototherapy or biologics for at least 3 months were excluded.
Barrea et al., 2016, Italy	50.0 (21.0–65.0)	29.0	Caucasian	ND	PsO	Patients attending a university hospital dermatology clinic. Controls matched based on age, sex and BMI.
De La Brassinne et al., 2016, Belgium	47.68 ± 16.12	45.7	ND	ND	PsO	Subjects attending a new outpatient psoriasis center recruited at random of their consultation.
Demirel et al., 2013, Turkey	37.8 ± 10.6	ND	ND	ND	PsO	Patients previously diagnosed with psoriasis by a trained dermatologist using biopsy. All patients defined as having mild to moderate psoriasis and receiving only topical therapy. Moderate psoriasis identified by PASI >10.
Diniz Mdos et al., 2016, Brazil	47.0	40.5	ND	47.0	PsO	Patients with psoriasis and without joint complaints, adult patients with clinical and/or histopathological diagnosis of psoriasis, without joint complaints, assisted at dermatology outpatient clinic. The control group comprised voluntary patients without psoriasis or any inflammatory disease, matched by sex and age.
Engin et al., 2014, Turkey	42.7	50.0	ND	13.6 ± 8.8	PsO	University psoriasis clinic. The diagnosis of psoriasis was based on clinical examination or histopathological examination in all patients.
Ferguson et al., 2019, UK	55.0 (43, 62 IQR)	63.0	ND	8.0 (2, 12.2 IQR)	PsA	Prospective, open label study of adults receiving apremilast as part of routine care for PsA and/or psoriasis.
Galluzzo et al., 2017, Italy	48.6 ± 16.5	36.6	ND	ND	PsO	Chronic plaque psoriasis patients, naive to biological drugs, treated with ustekinumab in the University dermatology clinic.
Galluzzo et al., 2018, Italy	32.1 ± 13.6	24.5	Caucasian	17.1 ± 11.5	PsO	Chronic plaque psoriasis patients, naive to biological drugs, treated with ustekinumab in the University dermatology clinic.
Ganguly et al., 2016, India	44.8 ± 12.8	ND	ND	0–20	PsO	Patients with chronic plaque psoriasis attending the dermatology outpatient clinic. Healthy controls who came for a routine health check to our hospital were included in the study.
Gonul et al., 2017, Turkey	43.2 ± 15.6	46.3	ND	14.6 ± 12.1	PsO	Patients with clinically and histopathologically diagnosed psoriasis, patients had been receiving systemic or topical treatment for the last month. Controls recruited from among patients with warts, tinea pedis, or melanocytic nevi without any inflammatory disease, and matched for age, sex and BMI.
Kofoed et al., 2012, Denmark	33.0 ± 8.6	ND	ND	15.9 ± 4.4	PsO	Eligible participants were anti-TNF–naïve patients with psoriasis recalcitrant to other systemic treatments and UV-B therapy. They also had a PASI or DLQI of >10.
Krajewska-Wlodarczyk et al., 2017, Poland	65.6 ± 5.9	100	ND	PsA 11.1 ± 8.9	PsA/PsO	Women diagnosed with PsA, treated at the rheumatology or dermatology clinic of the municipal hospital. The diagnosis of PsA was determined based on the CASPAR criteria. At least 12 months had passed since the last menstrual period.
				PsO 22.9 ± 12.9		
Leite et al., 2018, Brazil	ND	ND	ND	ND	PsA	ND
Renzo et al., 2011, Italy	PsA 42.2 ± 8.9	ND	ND	PsA 14.1 ± 11.2	PsA/PsO	Patients divided into two groups: 20 patients with PsO and 20 with PsA. Twelve patients received infliximab and 28 received etanercept.
	PsO 36.8 ± 8.4			PsO 15.0 ± 9.3		
Romani, Caixas et al., 2012, Spain	46.4 ± 17.3	61.3	ND	ND	PsO	PASI ≥ 10 evaluated by the same dermatologist. No systemic anti-psoriatic therapy (acitretin, ciclosporin, methotrexate or biologics), natural or artificial UV sources during the month prior to inclusion.
Romani, Caixas, Ceperuelo et al., 2012, Spain	ND	ND	ND	ND	PsO	Patients eligible for narrow-band UVB phototherapy recruited excluding the months of high solar irradiance. Controls without psoriasis matched to the patients’ characteristics. Patients with PsA were excluded, along with patients and controls with a list of chronic inflammatory diseases.
Tournadre et al., 2017, France	54.6 ± 11.0	63	ND	5.5 ± 6.8	PsA	ND
Toussirot et al., 2019, France	PsA 52.5 ± 11.7	51.2	ND	PsA 9.1 ± 6.7	PsA/PsO	Patients with PsO (plaque psoriasis) or PsA (CASPAR criteria) were evaluated. Each patient was paired to a control subject, recruited in the same outpatient population, and matched for sex, age and BMI.
	PsO 50.5 ± 12.8	26.9		PsA 18.1 ± 13.8		

ND: not defined.

### Clinical anthropometric assessment

All studies included clinical anthropometric data to varying degrees for overall measurement of participant shape and size. In all but one study [19], participants’ weight, height and BMI were measured. Several studies measured waist-to-hip ratio [29, 31–33], and one study supplemented this with skinfold thickness and abdominal circumference [31]. Only two studies commented on the attire of recruited subjects and attempted to reduce confounding through clothing by stipulating a dress code of light clothes and no shoes [24, 34]. These groups were assessed by a standard operator in a consistent manner. Very few studies incorporated participant information on smoking, alcohol, nutrition or physical activity, all of which are known to influence human metabolism [21, 24, 29, 34].

### Body composition

In general, 24 studies confirmed discrete biologic and body composition changes in patients with psoriatic disease, which correlated positively with other indicators of metabolic syndrome, including waist circumference, waist-to-hip ratio, weight, BMI, plasma concentrations of low-density lipoprotein (LDL)-cholesterol, leptin and apolipoprotein-B (apo-B).

One study failed to reveal statistically significant differences between psoriatic patients and controls with respect to maximal aerobic capacity, resting metabolic rate, pulmonary function tests, body fatness, body fat distributions and quality of life [31]. Moreover, not all studies found that these changes were correlated to either skin or joint disease activity [11, 19, 35, 36].

Three studies focused on muscle mass and reported levels of sarcopenia [18, 21, 22]. Aguiar et al. evaluated muscle mass by way of a muscle mass index (MMI: muscle/height^2^) and demonstrated reductions for spondyloarthritis with no significant variation between psoriatic arthritis and ankylosing spondylitis patients. They identified a significant difference in mean MMI between patients and controls (61.7% vs 43.3%: 7.65 ± 0.98 vs 8.25 ± 0.92; p = 0.001, OR = 5.23). MMI showed correlation with disease activity indices (BASDAI and BASMI) in the male AS patients only. However, their findings were not correlated with disease duration, function or radiological indices. A more recent study assessed sarcopenia and presarcopenia on the basis of European Working Group On Sarcopenia in Older People (EWGSOP) criteria and by using defined MMI cut-offs, and identified sarcopenia in 20.0% and presarcopenia in 25.7% of PsA patients. Their assessment did not include a sex- and age-matched control population [21].

### Measurement techniques

Ten techniques were used to describe body composition: BIA 10, DXA 6, CT 2, ultrasonography 1, transthoracic echocardiography 1 and other techniques 5 (including novel automated systems for measurement). Body composition outcomes by modality are shown in Table 4. Four studies [23, 24, 27, 34] measured Phase Angle (PhA), a direct measure of BIA, which researchers found was lower in the psoriatic patients. Barrea et al. [24] discovered that PhA was inversely associated with disease severity measured by Psoriasis Area Severity Index (PASI) and Dermatology Life Quality Index (DLQI); this was also found to be independent of BMI (*P* < 0.001), although this study included a low number of subjects.

**Table 4:** Body composition outcomes by modality.

	Study	Results	PsO vs HC
**Bioimpedance analysis (BIA)**	Barone et al., 2018	The prevalence of sarcopenia was about 20% in all the three rheumatic diseases, whereas presarcopenia was significantly different in RA, PsA and AS (*P* = 0.006).	**+**
	Barrea et al., 2015	PASI score was significantly associated with the % FM and CRP levels. At multiple regression analysis, the major predictor of PASI score were FM among BIA parameters, (r^2^ = 0.537, β = 0.740, *P* < 0.001.	**+**
	Barrea et al., 2016	PhAs ≤ 4.8° and ≤ 4.9° identified psoriatic patients who had the highest clinical severity and the lowest quality of life, respectively. Psoriatic patients presented small PhAs, with a novel association with clinical severity, quality of life and MetS. Among psoriatic patients, the PhA was the major index value for the diagnosis of MetS (OR 5.87, 95% CI 5.07–6.79) and was inversely associated with both PASI and DLQI, independently of BMI (*P* < 0.001).	**+**
	Demirel et al., 2013	Maximal aerobic capacity, resting metabolic rate, pulmonary function tests, body fatness, body fat distributions and quality of life were not statistically different between patients with psoriasis and controls in males and females.	ND
	Engin et al., 2014	Statistically significant differences were recorded in terms of weight (kg), body fat percentage, fat mass (kg), TBW percentage, metabolic age, visceral fat rating, BMI and degree of obesity among treated and untreated patients and control subjects.	**+**
	Galluzzo et al., 2017	Compared to men, women had several significantly higher bioelectrical impedance parameters including reactance, fat mass% and adipose tissue%. The values of adipose tissue were positively correlated only with patients’ age (p ¼ .021) and age at disease onset (p ¼ .0006) but not with disease severity. In addition, the use of BMI cut-offs allowed to categorize 36.7% of women and 19.2% of men as obese, while fat mass% showed that 53.3% of women and 48.1% of men were obese.	**+**
	Galluzzo et al., 2018	At baseline, psoriatic patients showed a lower BCM and mean PhA score. During ustekinumab treatment, the mean PhA and BCM scores increased with an improvement in psoriatic disease.	**+**
	Krajewska-Wlodarczyk et al., 2017	Sarcopenia, using ALM index and SMI, was diagnosed in 13.7% and 43.1% of PsA women, and in healthy women in 9% and 20.4%, respectively. In the group of PsA, sarcopenia was associated with a significant increase in the occurrence of disorders of bone mineralization (72.7% vs. 41.3% in patients without a decrease in muscle mass). There was no correlation between the loss of muscle mass, bone density, and activity of PsA.	**+**
	Romani, Caixas et al., 2012	With an identical body mass index, psoriatic patients showed a higher body fat content as calculated by electric bioimpedance, which correlated positively with waist circumference, and higher plasma concentrations of LDL-cholesterol, leptin and apo-B.	**+**
	Romani, Caixas, Ceperuelo et al., 2012	Ferritin, B12 and CRP decreased significantly after NB-UVB therapy. Vitamin D levels reached adequate levels after phototherapy; however, no relationship with PASI improvement was observed.	**+**
**Dual energy X-ray absorptiometry (DXA)**	Diniz Mdos et al., 2016	Dual energy X-ray absorptiometry was more capable of identifying obesity compared with BMI and WC both in psoriatic and control patients.	**+**
	Kofoed et al., 2012	Truncal fat percentage was negatively correlated with insulin sensitivity (r = −0.78; *P* = 0.01) and positively correlated with plasma leptin (r = 0.88; *P* = 0.002). After 12 weeks of therapy (infliximab = 5, adalimumab = 4), there were no significant changes in insulin sensitivity, fasting glucose, hemoglobin A1c, or C-peptide. Body fat increased by 6.5% and truncal fat increased by 11.4%. Leptin concentrations significantly decreased after anti-TNF treatment.	**+**
	Leite et al., 2018	The group on a hypocaloric diet + omega-3 supplementation (3 g/day) had significant weight loss (-1.79 ± 2.4; *P* = 0.004), as well as waist circumference (-3.28 ± 3.5; *P* = 0.001 and body fat (-1.2 ± 2.2; *P* = 0.006) reduction. There was no significant correlation between weight loss and disease activity improvement.	**+**
	Renzo et al., 2011	In both groups, a significant percentage change of body weight, fat, and lean mass, compared to baseline (PsO: body weight +3.8 ± 2.5%, fat mass 8.6 ± 10.5%, lean mass 1.9 ± 2.4%; PsA: body weight +3.7 ± 3.4%, fat mass 8.9 ± 17.3%, lean mass 2.9 ± 3.4%) was observed.	**+**
	Tournadre et al., 2017	Sarcopenia with a decrease in muscle mass and function was observed in 5 RA (7.8%), 1 SpA (1.7%) and 1 PsA (9.1%). Sarcopenia in terms of reduced SMI only (1) was not more frequent occurring in 5 RA (7.8%), 3 SpA (5.1%) and 1 PsA (9.1%).	**+**
	Toussirot et al., 2019	Total fat mass was increased in PsO but not in PsA. Android fat and visceral fat were found higher in PsO (p< 0.05), while the fat mass measurements did not differ between the patients with PsA and controls. WC was higher in patients with PsO compared to their controls. Leptin, leptin/fat mass ratio, and total adiponectin were elevated in PsA. Insulin levels and HOMA were increased in both PsO and PsA groups. In patients with PsO, android and visceral fat were correlated with SCORE (r = 0.3; *P* = 0.02 and r = 0.6; *P* < 0.0001 respectively).	**+**
**Ultrasonography**	Gonul et al., 2017	AFI was not associated with psoriasis in subjects with metabolic syndrome (*P* = 0.495) or with Psoriasis Area and Severity Index (r = 0.123; *P* = 0.443).	**+**
**Transthoracic echocardiography**	Akyildiz et al., 2014	EFT was significantly associated with MS (*P* < 0.01), age (*P* < 0.05) and female gender (*P* < 0.05) in psoriasis patients only.	**+**
**Computed tomography (CT)**	Balci et al., 2010	The mean VFA value and VFA/SFA ratio of the psoriasis patients were significantly higher compared with control patients (123.4 ± 80.3 vs. 81.2 ± 59.8 cm^2^ and 0.734 ± 0.593 vs. 0.491 ± 0.336; *P* = 0.005 and *P* = 0.017, respectively). Fasting blood sugar levels were significantly higher in psoriasis patients, compared with control patients (101.8 ± 43.5 vs. 83.4 ± 9.1 mg/dl; *P* = 0.005). Multiple linear regression analysis indicated that waist-to-hip ratio, age, body weight, the presence of psoriasis and metabolic syndrome were significantly associated with VFA.	**+**
	Balci et al, 2014	Mean EFA in patients with psoriasis was significantly higher than in controls (13.8 ± 8.4 vs. 97 ± 6.4 cm^2^, respectively; *P* = 0.02), but mean CACS did not differ significantly between the two groups (55.2 ± 65.4 vs. 27.8 ± 29.3; *P* > 0.05).	**+**
**Other**	Aguiar et al., 2014	62% of patients had sarcopenia and there was a significant difference in MMI between patients and controls (7.65 ± 0.98 vs 8.25 ± 0.92; *P* = 0.001, OR = 5.23). In male patients, there was a statistically significant moderate negative correlation between MMI and BASDAI and BASFI (*P* = -0.536 and *P* = -0.445). No other significant correlations were identified.	**+**
Mean muscle index (MMI)			
Body fat (%)	Andreevskaia et al., 2013	Mean body fat percentage 31.84 + 8.58% (36.60 + 7.32% in women and 27.17 + 7.13% in men) exceeding the maximum of a normal population by a mean absolute percentage of 3.74% (corrected = 13.30%).	**+**
Body fat percentage (%)	De La Brassinne et al., 2016	These results confirm the increased frequency of obesity in patients with psoriasis and are obtained by a fully automated conversational system usable by the patient.	**+**
Lipid accumulation product (LAP) index, a measure of visceral fat	Ganguly et al., 2016	A statistically significant difference (*P* < 0.001) was observed between the LAP index of controls (23.79 ± 13.02) and that of psoriasis patients (46.42 ± 27.2). LAP index was significantly higher (*P* = 0.028) in the moderate-to-severe psoriasis group (56.02 ± 30.09) as compared to the mild psoriasis group (36.82 ± 20.49).	**+**
MRI of abdominal fat distribution	Ferguson et al., 2019	Mean weight loss after 3 and 6 months apremilast treatment was -1.4kg (95% CI -2.1, -0.6, *P* = 0.001) and -2.2kg (95% CI -3.1, -1.4, *P* < 0.001), respectively. >5% weight loss was achieved in 6.1% (3/49) and 21.4% (9/42) after 3 and 6 months of treatment, respectively.	**+**

*P*-values are reported when provided in the published studies; +: favors controls.

AFI: abdominal fat index; ALM: appendicular lean mass; AS: ankylosing spondylitis; BCM: body cellular mass; BIA: bioelectric impedance analysis; BMI: body mass index; CACS: coronary artery calcium score; CT: computed tomography; DXA: dual energy X-ray absorptiometry; EFA: epicardial fat area; EFT: epicardial fat thickness; FM: fat mass; FMI: fat muscle index; HC: healthy controls; HOMA: Homeostatic Model Assessment; LAP: lipid accumulation product; MetS: metabolic syndrome; MMI: mean muscle index; MRI: magnetic resonance imaging; ND: no difference; NR = not recorded; PhA: data on phase angle; PsA: psoriatic arthritis; PsO: psoriasis; RA: rheumatoid arthritis; SCORE: Systematic Coronary Risk Evaluation; SMI: skeletal muscle index; SFA: subcutaneous fat area; SpA: spondyloarthritis; TBW: total body water; VFA: visceral fat area; WC: waist circumference.

The following devices were used to derive body composition data for analysis in the 10 included BIA studies: BIA 101, Akern srl, Pontassieve, Florence, Italy [21]; single-frequency 50 kHz BIA 101 RJL, Akern Bioresearch, Florence [23, 24, 27, 34]; Bodystat 1500, Bodystat Ltd., Douglas, Isle of Man, UK [31]; Tanita SC-330 Body Composition Analyzer, Tanita Corp., Tokyo, Japan [37]; InBody 170, Biospace, South Korea [11]; Tanita TBF300, Tanita Corporation, Tokyo, Japan [35, 36].

All studies bar one [31] demonstrated less favorable body composition data in psoriasis patients compared to controls.

### Body composition associations

Balci et al. [32] identified increased visceral fat area (VFA) using CT in psoriasis patients vs. controls. Multiple linear regression analysis in all study subjects indicated that VFA was significantly associated with waist:hip ratio, age, body weight and presence of metabolic syndrome, though not PASI score, duration or type of disease, smoking habit and therapies. A second study from this group studied epicardial fat [33], in which epicardial fat area (EFA) and coronary artery calcium scoring (CACS) were measured in patients with psoriasis and controls: EFA was significantly associated with CACS, waist circumference and age in the psoriasis patients only. No clinical features or laboratory findings were coupled to EFA. Barone et al. [21] showed age, CRP and disability were associated with sarcopenia, whereas the type of rheumatic disease (RA, PsA or AS), gender, calorie and protein intake, physical activity level, biologic treatment, duration of disease and ESR were not associated with an increased risk of sarcopenia.

### Effect of treatments

Six studies reported on cardiometabolic and body composition improvements on patient profiles after receiving specific interventions: hypocaloric diet with omega-3 supplementation [19], phosphodiesterase-4 (PDE4) inhibition with apremilast [20], narrowband ultraviolet (NB-UVB) therapy [35], anti-TNFα administration with infliximab or etanercept [29], anti-TNFα administration with infliximab or adalimumab [28] and anti-IL-12/23 administration with ustekinumab [23].

### Assessment of bias

The risk of bias quality assessment of included studies is presented in Table 5. Six of the studies, whilst identifying as case-control, were reassigned as cross-sectional design on account of their methodology [22, 24, 36, 38–40]. Nine studies did not include a control group [18, 20, 21, 23, 25–29]. Several studies did not fully specify how their participants were recruited [21, 26, 29, 31] and there was uncertainty about this information from an additional four studies [11, 18, 20, 28]. The sample size was small (< 40) for a number of studies [28, 31, 33, 38]. Overall, four studies were regarded to be at high risk of bias [18, 21, 26, 29], two at medium risk of bias [28, 31] and 19 at low risk of bias [11, 19, 20, 22–25, 27, 30, 32–41] using the ROBINS-I tool.

**Table 5:** Risk of bias assessment.

	Was the sample representative of the target population?	Were cases recruited in an acceptable way?	Did the study include a control group?	Were the controls selected in an acceptable way?	Was there matching of controls?	Was the sample size based on pre-study considerations of statistical power?	Were the groups treated equally?	Was there appropriate statistical analysis?	Were important confounding factors identified and accounted for?	Will the results help locally?
Aguiar et al., 2014	Y	Y	Y	Y	N	N	Y	Y	Y	Y
Akyildiz et al., 2014	Y	Y	Y	Y	N	N	Y	Y	Y	Y
Andreevskaia et al., 2013	Y	Y	N	N/A	N/A	N	N/A	N	?	Y
Balci et al., 2010	Y	Y	Y	Y	Y	Y	Y	Y	?	Y
Balci et al., 2014	Y	Y	Y	Y	Y	Y	Y	Y	?	Y
Barone et al., 2018	N	N	N	N/A	N/A	N	N/A	N	N	N
Barrea et al., 2015	Y	Y	Y	Y	Y	N	Y	Y	Y	Y
Barrea et al., 2016	Y	Y	Y	Y	Y	Y	Y	Y	Y	Y
De La Brassinne et al., 2016	?	N	N	N/A	N/A	N	N/A	Y	N	N
Demirel et al., 2013	?	N	Y	?	N	N	?	Y	N	Y
Diniz Mdos et al., 2016	Y	?	Y	Y	Y	N	Y	Y	N	Y
Engin et al., 2014	Y	Y	Y	?	N	Y	Y	Y	Y	Y
Ferguson et al., 2019	Y	?	N	N/A	N/A	N	N/A	Y	?	Y
Galluzzo et al., 2017	Y	Y	N	N/A	N/A	N	N/A	Y	Y	Y
Galluzzo et al., 2018	?	Y	N	N/A	N/A	N	N/A	Y	Y	Y
Ganguly et al., 2016	Y	Y	Y	Y	N	Y	Y	Y	N	Y
Gonul et al., 2017	?	Y	Y	Y	Y	N	Y	Y	N	Y
Kofoed et al., 2012	?	?	N	N/A	N/A	N	N/A	Y	N	N
Krajewska-Wlodarczyk et al., 2017	Y	?	Y	?	Y	N	?	Y	Y	Y
Leite et al., 2018	Y	Y	Y	?	?	N	Y	Y	Y	N
Renzo et al., 2011	N	N	N	N/A	N/A	N	N/A	N	N	N
Romani, Caixas et al., 2012	Y	Y	Y	Y	Y	N	Y	Y	Y	Y
Romani, Caixas, Ceperuelo et al., 2012	Y	Y	Y	Y	Y	N	Y	Y	Y	Y
Tournadre et al., 2017	?	?	N	N/A	N/A	N	N/A	Y	N	Y
Toussirot et al., 2019	Y	Y	Y	Y	Y	N	Y	Y	Y	Y

N: no; N/A: not applicable; Y: yes;?: unclear.

### Additional analyses

Sensitivity analysis was not feasible due to clinical heterogeneity preventing meta-analysis.

## Discussion

To our knowledge, this is the first systematic review examining the relationship between psoriatic disease and whole-body composition as a distinct entity from metabolic syndrome. Strengths of the review include use of a high degree of rigor in its search strategy and screening procedures and using relevant standards for performing systematic reviews. We tried to reflect the complete picture of both published and unpublished literature on this topic including smaller studies and conference proceedings. Evidently, the last decade has seen an exciting expansion of interest in the development and validation of new modalities for the assessment of body composition. Our study provides evidence for a relationship between certain body composition phenotypes and the occurrence of psoriasis, including higher overall body fat, visceral fat and sarcopenia, that is similar, yet distinct, from the metabolic syndrome. Several aspects of body composition, specifically the amount and distribution of body fat and lean mass, are now understood to be important health outcomes in adults and should form an important part of the ongoing clinical assessment of patients with psoriasis. However, the issue of whether body compartment distribution is a result of severe psoriasis or a causative factor in its development remains contentious. It is likely that novel systems will eventually supplement less sophisticated bedside measurements and influence key aspects of risk assessment, prognostication and management.

We found an increased prevalence of body composition derangements in psoriatic patients compared with controls. As expected, parameters associated with obesity, such as weight, body fat percentage, fat mass and degree of obesity, were higher in the psoriasis groups than in the control groups, irrespective of therapy. Such an association between obesity and psoriasis has been well documented, first described in 1986 [42]. Traditional epidemiologic studies have focused on weight or BMI to define obesity rather than altered body composition. We found conflicting data on the association between psoriasis severity, such as PASI, and body composition parameters, indicating that a causal link is not definitive. Previous studies have alluded to a dose-response relationship between psoriasis severity and metabolic syndrome [43], supported by translational studies showing T-helper cell cytokine upregulation in the blood and skin of psoriasis patients, leading to effects on lipid metabolism and insulin resistance [44]. Furthermore, our data exemplify how there is insufficient evidence to infer that PsA carries a higher metabolic burden than PsO; thus, more studies are required in this area to distinguish distinct body composition and metabolic profiles of subtypes of psoriatic disease.

In this review, treatment with anti-IL-12/23 or PDE4 inhibitors was associated with more favorable body composition profiles than anti-TNFα treatments, findings which mirror previous observations of increases in BMI seen with this drug class [45, 46]. IL-17, one of the key proinflammatory cytokines in psoriasis, mechanistically links inflammation with insulin resistance and adipocyte dysfunction [47]. IL-17A producing cells are thought to be pathogenic in driving inflammation in obesity and progression of obesity-related inflammatory diseases, suggesting that causality between psoriasis and adipogenesis is likely to be bidirectional [48]. From this perspective, there are likely to be therapeutic implications of targeting proinflammatory factors like IL-17 or IL-12/23 in metabolic dysfunction associated with psoriatic disease.

Our review highlights that there are no data comparing and validating body composition techniques in a psoriatic population; therefore, drawing conclusions about the most precise or reliable technique is not possible. Due to its non-invasiveness, low cost and portability, it is easy to appreciate how BIA was adopted by most researchers. The technique relies on the assumption that the volume of fat-free tissue will be proportional to the electrical conductivity of the body. It employs a small electric current to measure the resistance and reactance at difference frequencies against various tissues in the body e.g. lipid has a high resistance to the flow of current, therefore shows a high impedance reading, whereas muscle, which stores most of our body water, has lower impedance. BIA assessment tools have been considered a promising approach for the quantitative measurement of tissue characteristics over time, as well as demonstrating the direct relativity between fluctuations in body composition and prognosis, clinical condition and quality of life [49]. The technique offers reliable data on body composition provided that suitable (i.e. age-, sex- and population-specific) equations for the calculation of body compartments are applied [50]. However, a major limitation of this technique pertains to measurement discrepancies between devices from different manufacturers and the lack of internationally recognized standard reference values.

PhA is thought to be one of the most clinically relevant parameters of BIA. It is defined as the ratio of resistance (intracellular and extracellular resistance) to reactance (cell membrane-specific resistance), expressed as an angle. It is considered an indicator of cellular health, where higher values reflect cell membrane integrity and better cell function. In healthy populations, increasing age bestows a lower PhA due to a reduction in reactance and a parallel loss of muscle mass and an increase in resistance due to the declining proportion of body water at the expense of fat mass. In disease, PhA is often reduced because of infection, inflammation or disease-specific determinants [51]. Recent studies have reported that PhA in humans follows a linear relationship with cellular health and can be considered a prognostic tool in certain medical disorders, including cancer, cirrhosis and diabetes mellitus [24, 52–56]. It is important to note that not all BIA devices can detect phase-sensitive impedance variation that can be used for assessment of phase angle.

It is clear that there is no single method of body composition measurement that allows for the delineation of all tissues and organs, and there are pros and cons of all techniques. The seemingly unsophisticated measurements of skin thickness, BMI and waist circumference can provide simple longitudinal assessments of fatness and metabolic risk despite their poor accuracy and inability to differentiate fat and lean masses. The value of any approach in supporting clinical practice is enhanced by the availability of reference data. Recent developments include MRI for fat distribution.

### Limitations

This systematic review should be interpreted in the context of the reported studies which were heterogeneous in several aspects. Firstly, the observational studies recruited different extents and subtypes of psoriatic disease, some with associated arthritis, and measured different aspects of body composition, making definitive conclusions problematic. Secondly, there was poor matching of patients and controls across all studies and little consideration for the potential confounding effects of key determinants of metabolism, e.g. physical activity, age and smoking. Finally, BIA-estimated percentage of body fat varies greatly with population and age and is directly and closely related to various health outcomes such as cardiovascular diseases. Despite its prognostic potential, BIA has not been validated in population studies or clinical practice due to lack of normal population reference limits for comparison and is also influenced by other factors such as age, sex and race [57]. We aimed to present a wide variety of research endeavors in this upcoming field though we recognize that we may not have captured smaller conference proceedings or non-English publications.

### Further considerations

We suggest that body composition indices should be analyzed in more detail using a broader range of techniques and imaging systems across the clinical spectrum of psoriatic patients in order to generate validated methods of assessment, particularly with regards to the prognostic ability of BIA and PhA. Further studies are needed to address the discrepancies in bioimpedance parameters within body compartments and between different devices and the deviation from health to disease. We hope that future studies will reveal insights into drug-specific alterations in body composition profiles in psoriatic disease, enabling clinicians to practice more stratified medicine and treat more effectively the metabolic components of patients’ disease that are so often neglected in clinical practice and associated with worse outcomes.

## Conclusions

In conclusion, this study provides evidence for a relationship between certain body composition phenotypes and the occurrence of psoriasis, including higher overall body fat, visceral fat and sarcopenia that are similar, albeit distinct, from the metabolic syndrome. Several aspects of body composition, specifically the amount and distribution of body fat and lean mass, are now understood to be important health outcomes in adults and should form an important part of the ongoing assessment of patients with psoriasis. However, the issue of whether body compartment distribution is a result of severe psoriasis or a causative factor in its development remains contentious. It is hoped that novel systems will eventually supplement less sophisticated bedside measurements and influence key aspects of risk assessment, prognostication and management.

## Supporting information

S1 Fig PRISMA 2009 checklist. (TIF)

S2 Fig Full Medline and Embase search strategy. (TIF)

S3 Fig Data extraction form. (TIF)
