# Supplementary figures and images for: Psoriatic disease and body composition: A systematic review and narrative synthesis
Source: PLoS One. 2020 Aug 13;15(8):e0237598. doi: 10.1371/journal.pone.0237598 (PMC7425946; doi:10.1371/journal.pone.0237598)

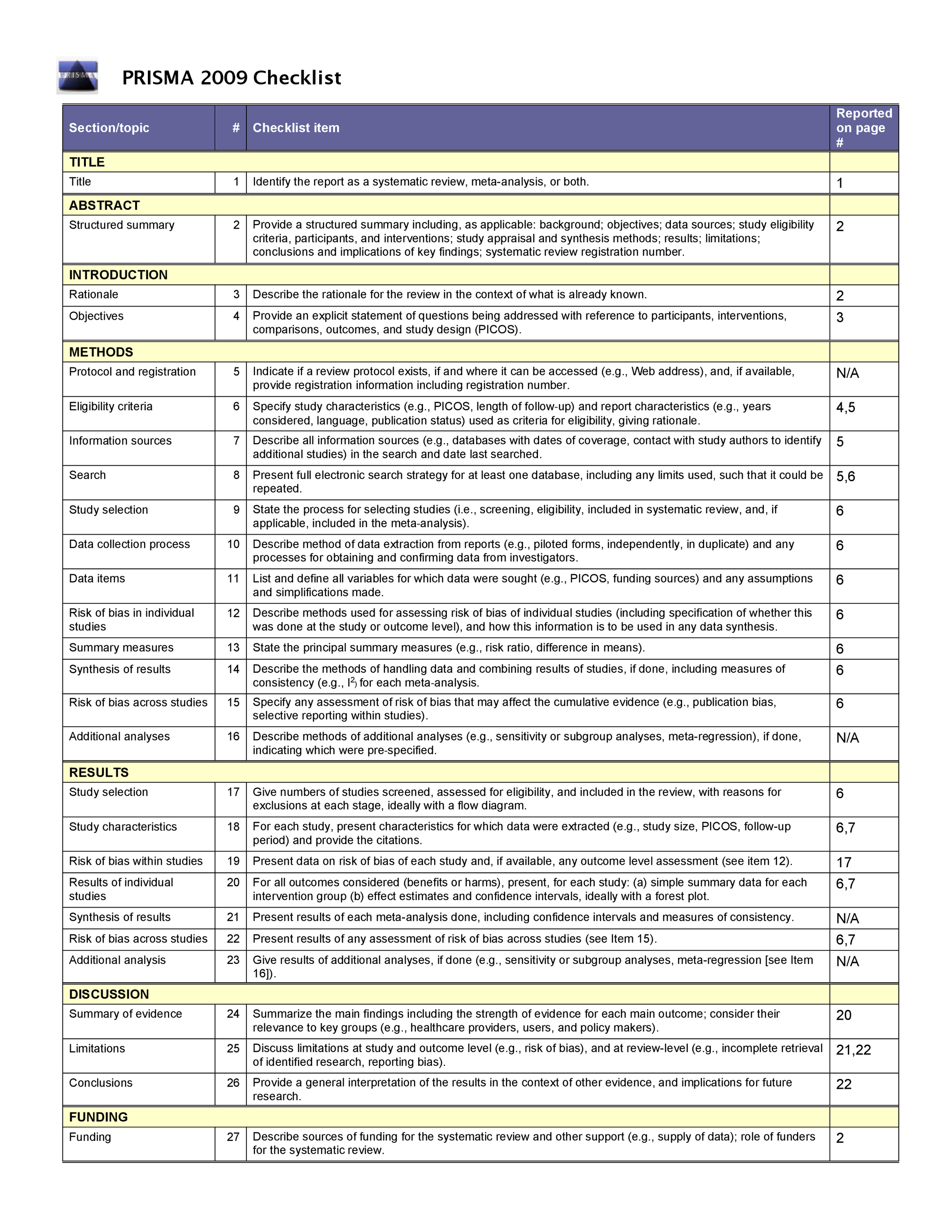

Supplement: S1 Fig — (TIF) [file pone.0237598.s001.tif]

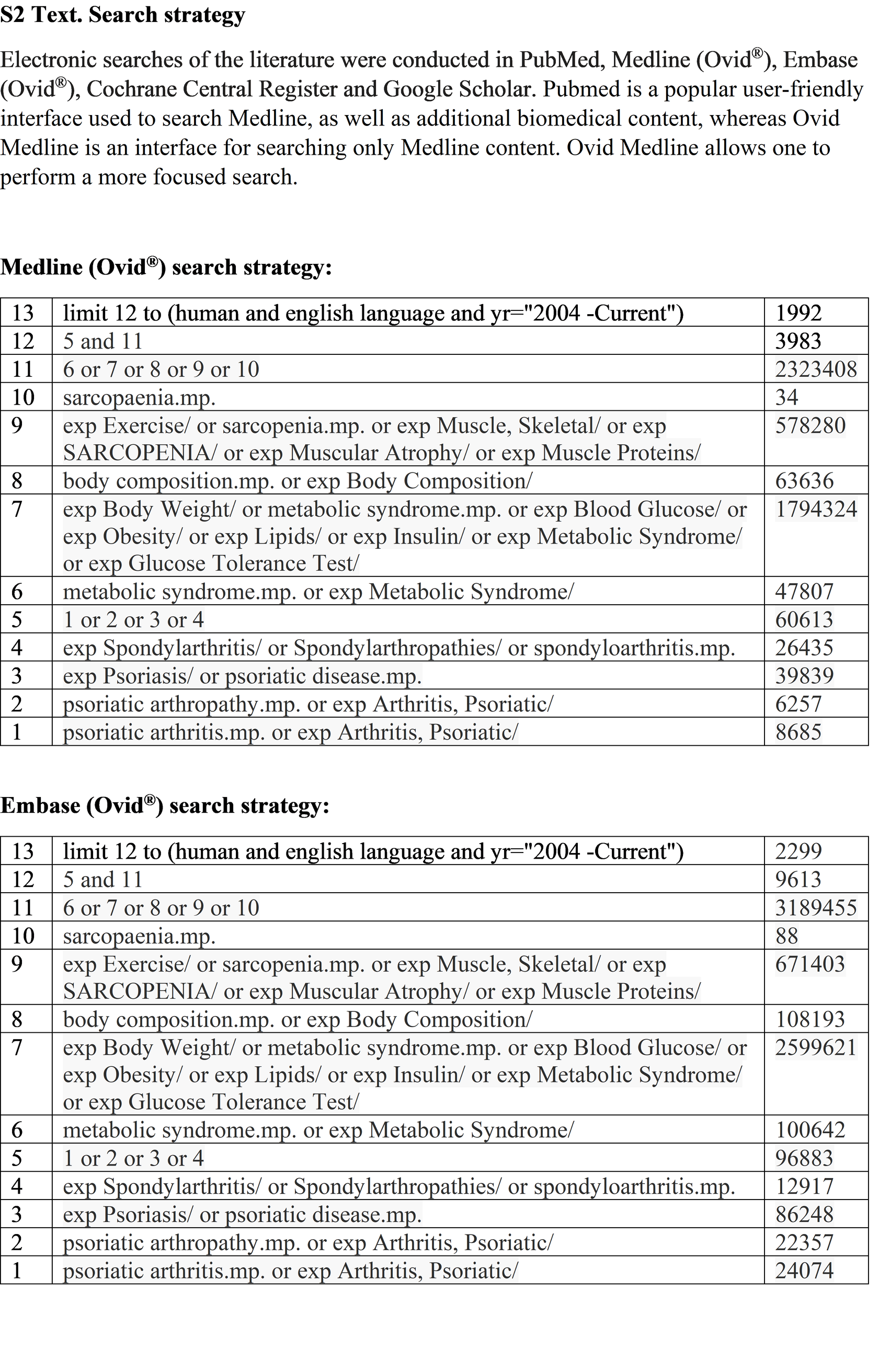

Supplement: S2 Fig — (TIF) [file pone.0237598.s002.tif]

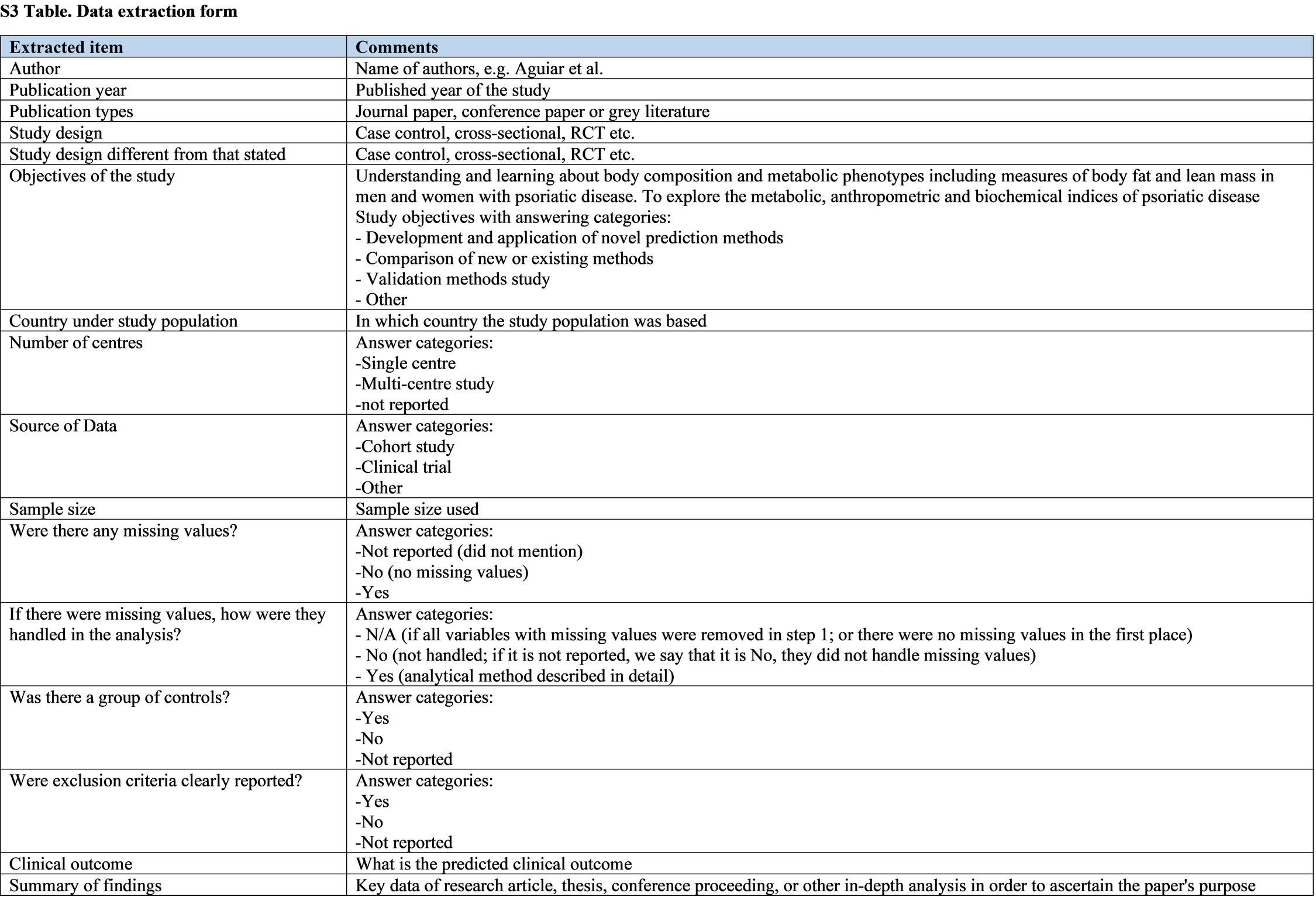

Supplement: S3 Fig — (TIF) [file pone.0237598.s003.tif]
